# Incidence and prognostic impact of HER2‐positivity loss after dual HER2‐directed neoadjuvant therapy for HER2+ breast cancer

**DOI:** 10.1002/cam4.5817

**Published:** 2023-03-27

**Authors:** Alexis LeVee, Kellie Spector, Brigid Larkin, Felipe Dezem, Jasmine Plummer, Farnaz Dadmanesh, Sujata Patil, Heather L. McArthur

**Affiliations:** ^1^ Department of Medicine Cedars‐Sinai Medical Center Los Angeles California USA; ^2^ Department of Medical Oncology and Therapeutics Research City of Hope Comprehensive Cancer Center Duarte California USA; ^3^ Department of Medicine Olive View‐UCLA Medical Center Los Angeles California USA; ^4^ Center for Bioinformatics and Functional Genomics, Department of Biomedical Sciences Cedars‐Sinai Medical Center Los Angeles California USA; ^5^ Department of Pathology Cedars‐Sinai Medical Center Los Angeles California USA; ^6^ Department of Quantitative Health Sciences Cleveland Clinic Taussig Cancer Institute Cleveland Ohio USA; ^7^ Department of Medicine University of Texas Southwestern Dallas Texas USA

**Keywords:** breast cancer, discordance, dual, HER2, neoadjuvant therapy

## Abstract

**Background:**

Loss of HER2 “positivity” can occur in patients with residual disease after neoadjuvant treatment, but the incidence of HER2‐positivity loss after neoadjuvant dual HER2‐targeted treatment plus chemotherapy, the current standard‐of‐care for most early stage HER2‐positive breast cancers, is not well described. Previous studies that report the HER2 discordance rate after neoadjuvant treatment also do not include the novel HER2‐low category. In this retrospective study, we determine the incidence and prognostic impact of HER2‐positivity loss, including the evolution to HER2‐low disease, after neoadjuvant dual HER2‐targeted therapy with chemotherapy.

**Methods:**

Clinicopathologic data for patients with stage I‐III HER2+ breast cancer diagnosed between 2015 and 2019 were reviewed in this single institution retrospective study. Patients who received dual HER2‐targeted treatment with chemotherapy were included, and HER2 status before and after neoadjuvant therapy was interrogated.

**Results:**

A total of 163 female patients were included in the analysis with a median age of 50 years. A pathologic complete response (pCR as defined by ypT0/is) was achieved in 102 (62.5%) of 163 evaluable patients. Among the 61 patients with residual disease after neoadjuvant therapy, 36 (59.0%) had HER2‐positive and 25 (41.0%) had HER2‐negative residual disease. Of the 25 patients with HER2‐negative residual disease, 22 (88%) of patients were classified as HER2‐low. After a median follow‐up of 3.3 years, patients who retained HER2‐positivity after neoadjuvant treatment had a 3‐year IDFS rate of 91% (95% CI, 91%–100%), while patients who lost HER2‐positivity had a 3‐year IDFS rate of 82% (95% CI, 67%–100%).

**Conclusion:**

Almost half of patients with residual disease following neoadjuvant dual HER2‐targeted therapy plus chemotherapy lost HER2‐positivity. The loss of HER2‐positivity may not confer negative prognostic impact, although the results were limited by short follow‐up time. Further research on the HER2 status after neoadjuvant treatment may help guide treatment decisions in the adjuvant setting.

## Graphical abstract

The incidence of loss of HER2‐positivity after neoadjuvant dual HER2‐targeted therapy with chemotherapy is not well described, but may affect adjuvant systemic therapy recommendations. In this study, almost half of the patients with residual disease after neoadjuvant dual HER2‐targeted treatment plus chemotherapy lost HER2‐positivity at the time of surgery. Of these patients, almost all were classified as HER2‐low. The loss of HER2‐positivity may not confer negative prognostic impact, although the results were limited by short follow‐up time.

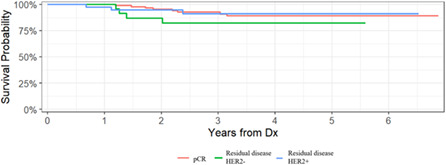

## 1 INTRODUCTION

Human epidermal growth factor receptor 2 (HER2) is a transmembrane receptor tyrosine kinase that is amplified/overexpressed in approximately 15% of breast cancers.^1^ With the development of trastuzumab, a monoclonal antibody that binds to the extracellular domain of the HER2 receptor, pathologic complete response (pCR), invasive disease‐free survival (IDFS), and overall survival (OS) outcomes have drastically improved for women with HER2‐positive breast cancer.^1^, ^2^, ^3^ Other HER2‐targeted agents have since been developed, including pertuzumab, a humanized monoclonal antibody that inhibits HER2 dimerization. Because trastuzumab and pertuzumab bind to separate epitopes on the HER2 receptor, they have complementary mechanisms of action.^4^ The phase 2 NeoSphere neoadjuvant trial demonstrated improved pCR rates when pertuzumab was combined with trastuzumab in the neoadjuvant setting and ultimately led to the United States Food and Drug Administration (FDA) accelerated approval of pertuzumab in combination with trastuzumab and chemotherapy in the neoadjuvant setting.^4^

In order to guide treatment decisions with HER2‐targeted therapies, correct histopathological assessment is necessary to determine HER2‐positivity. According to the 2018 American Society of Clinical Oncology (ASCO)/College of American Pathologists (CAP) guidelines, HER2 status is assessed by immunohistochemistry (IHC), which measures HER2 protein levels on the tumor cell membrane, and/or by fluorescence in situ hybridization (FISH), which measures HER2 gene amplification.^5^ Discordance of HER2 status in paired biopsies can occur in both the early and metastatic settings in breast cancer.^6^ In the neoadjuvant setting, HER2 discordance refers to the loss of HER2 expression or amplification in residual tumor found at surgery compared with the initial biopsy after intervening neoadjuvant HER2‐directed therapy. Although the specific mechanisms leading to HER2 discordance are unclear, tumor heterogeneity leading to clonal selection of HER2‐negative clones and the development of resistance mechanisms leading to HER2 loss have been implicated. Technical errors related to sampling and HER2 amplification methodology may also result in HER2 discordance. Studies have shown that the incidence of HER2 discordance after neoadjuvant trastuzumab with chemotherapy ranges from 7% to 47%, but evidence is mixed regarding whether the loss of HER2‐positivity after neoadjuvant systemic treatment impacts disease‐free recurrence and survival.^7^, ^8^, ^9^, ^10^, ^11^, ^12^, ^13^, ^14^, ^15^, ^16^, ^17^, ^18^, ^19^, ^20^ The impact of HER2‐positivity loss with neoadjuvant dual HER2‐targeted therapy with chemotherapy, the current standard‐of‐care, is not well described.

HER2‐negativity has been traditionally classified as IHC 1+ or 2+ in the absence of HER2 gene amplification by FISH; however, a new nomenclature has been proposed for HER2‐negative breast cancers that exhibit low HER2 expression, namely HER2‐low and HER2‐ultralow.^21^ HER2‐low refers to tumors with IHC 1+ or 2+ with FISH negative, while HER2‐ultralow refers to IHC 0. This new nomenclature has been proposed due to the subset of HER2‐low breast cancer patients who have benefited from the novel antibody–drug conjugates (ADCs), namely trastuzumab deruxtecan (T‐DXd) which was shown to be effective for HER2‐low metastatic breast cancer in the phase 3 DESTINY‐Breast04 trial.^22^ T‐DXd is an ADC consisting of a humanized trastuzumab covalently linked to a topoisomerase I inhibitor payload through a tetrapeptide‐based cleavable linker. Due to the potent cytotoxic payload (drug‐to‐antibody ratio 8:1), T‐DXd has a bystander effect which allows it to kill neighboring cells with HER2‐low expression.^22^ Given the efficacy of T‐DXd in HER2‐low metastatic breast cancer, the question remains whether patients with HER2‐low disease following neoadjuvant therapy will derive similar benefit from T‐DXd. The phase 3, randomized DESTINY‐Breast05 study (ClinicalTrials.gov identifier: NCT04622319) investigating T‐DXd versus trastuzumab emtansine (T‐DM1) as adjuvant therapy in patients with HER2‐positive early breast cancer is currently recruiting and may shed light on this issue. However, current guidelines recommend the use of adjuvant T‐DM1, an ADC consisting of trastuzumab and the cytotoxic agent emtansine (DM1), for patients with HER2‐positive early breast cancer who have residual disease after neoadjuvant therapy based on the phase 3 KATHERINE study, which showed benefit of adjuvant T‐DM1 compared to trastuzumab, regardless of HER2 status of the residual disease.^23^ A change in HER2 status after neoadjuvant therapy currently does not have therapeutic implications, but whether patients with HER2‐low and HER2‐ultralow disease may achieve greater benefit from ADCs or other therapeutic agents is currently unknown.

The aim of this study was to assess the incidence and prognostic impact of HER2‐positivity loss, including the evolution to HER2‐low disease, after dual HER2‐targeted therapy with chemotherapy in the neoadjuvant setting.

## 2 PATIENTS AND METHODS

**FIGURE 1:**
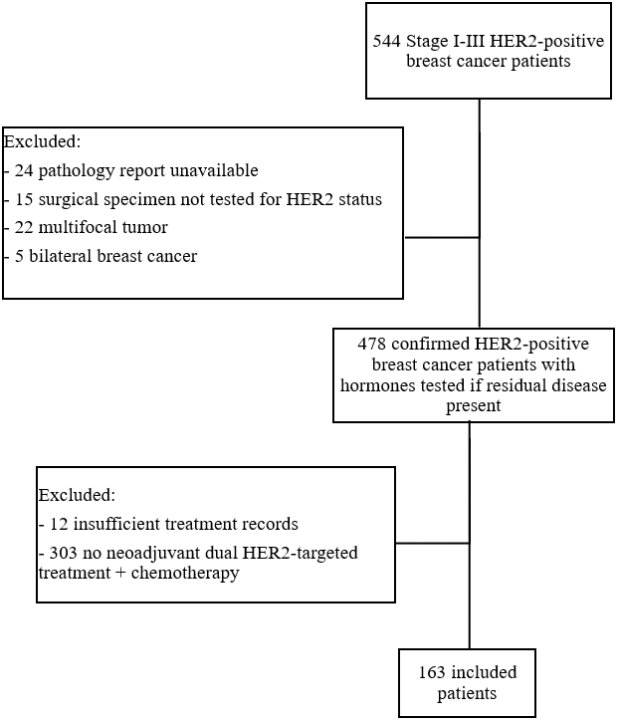
Consort diagram.

Patients with stage I‐III HER2‐positive breast cancer diagnosed at Cedars‐Sinai Medical Center between January 1, 2015 and December 31, 2019 were identified through an institutional database and electronic medical records were reviewed. Patients were included in the analysis if matched biopsy and surgery pathology reports were available, and HER2 status in the breast was reported if residual disease was present. Pathology reports from outside institutions were included if they met these requirements, and centralized HER2 testing was not consistently performed given the retrospective nature of the study. Bilateral tumors and multicentric tumors with heterogenous hormone receptor profiles were excluded due to the anticipated differences in response to HER2‐targeted treatment. Of the 478 HER2‐positive breast cancer patients remaining, 303 patients were excluded due to not having received neoadjuvant dual HER2‐targeted treatment plus chemotherapy and 12 patients were excluded due to insufficient treatment records or follow‐up. A total of 163 patients were ultimately evaluable (Figure 1).

HER2 was considered positive based on immunohistochemistry (IHC) score of 3+ and/or fluorescence in situ hybridization (FISH) ratio of ≥2.0 for the number of HER2 gene copies to the number of signals for CEP17. Tumors that were FISH negative by HER2/CEP17 ratio <2.0 with an average copy number ≥6 were excluded due to the limited number of patients who met this criteria (4) and the change in the American Society of Clinical Oncology (ASCO)/College of American Pathologists (CAP) guidelines from the 2013 and 2018 update regarding this clinical scenario. Pathologic complete response (pCR) was defined as ypT0/is and no lymph node metastasis. This study was approved by Cedars‐Sinai Medical Center institutional review board.

Patient and clinical characteristics were compared using two sample tests (Wilcoxon rank‐sum test for continuous variables and Fisher exact test or Chi‐square test for categorical) between two groups defined by residual disease status and in a second analysis by residual disease HER2 status. Invasive disease‐free survival (IDFS) was defined as “date of surgery” to “date of last clinical note documenting disease status” or “recurrence date” whichever presented first. Overall survival (OS) was defined as the “date of surgery” to “date of last clinical note documenting disease status.” Survival probability distribution was calculated using the Kaplan–Meier method. Two‐sided *p*‐values were considered significant if they were <0.05. All analyses were conducted in R v4.04.^24^

## 3 RESULTS

Clinicopathologic data and neoadjuvant treatment regimens by residual disease status are summarized in Table 1. A total of 163 female patients were identified and included in the analysis with a median age of 50 years. The majority of patients had ductal histology (95%), clinical T2 disease (58%), and nuclear grade 3 tumors (60%). Almost half (49%) had negative lymph nodes, 66% had estrogen receptor (ER) positive disease, 47% had progesterone receptor (PR) positive disease, and 79% had IHC 3+ tumors. Docetaxel, carboplatin, and trastuzumab with pertuzumab (TCHP) was the most commonly administered neoadjuvant regimen (85%) followed by paclitaxel, trastuzumab, and pertuzumab (THP, 8%).

**TABLE 1:** Characteristics of patients, tumors, and neoadjuvant regiments overall and by pCR status.

Characteristics	Whole cohort	By pCR status		
	*N* = 163^a^	*N*, *N* = 61^b^	*Y*, *N* = 102^b^	*p*‐value^c^
Age at diagnosis	51 (13) 50 [21 81]	49 (13) 48 [26 81]	52 (13) 51 [21 80]	0.10
CB histology				
Ductal	155/163 (95%)	57/61 (93%)	98/102 (96%)	
Lobular	4/163 (2.5%)	2/61 (3.3%)	2/102 (2.0%)	
Mixed	3/163 (1.8%)	1/61 (1.6%)	2/102 (2.0%)	
Unspecified	1/163 (0.6%)	1/61 (1.6%)	0/102 (0%)	
Clinical T classification				0.10
1	22/163 (13%)	7/61 (11%)	15/102 (15%)	
2	94/163 (58%)	32/61 (52%)	62/102 (61%)	
3	41/163 (25%)	17/61 (28%)	24/102 (24%)	
4	6/163 (3.7%)	5/61 (8.2%)	1/102 (1.0%)	
Clinical N classification				0.10
0	80/162 (49%)	23/60 (38%)	57/102 (56%)	
1	63/162 (39%)	30/60 (50%)	33/102 (32%)	
2	8/162 (4.9%)	2/60 (3.3%)	6/102 (5.9%)	
3	11/162 (6.8%)	5/60 (8.3%)	6/102 (5.9%)	
NX	1	1	0	
CB nuclear grade				0.013
2	54/163 (33%)	29/61 (48%)	25/102 (25%)	
2.5	7/163 (4.3%)	3/61 (4.9%)	4/102 (3.9%)	
3	98/163 (60%)	28/61 (46%)	70/102 (69%)	
Unknown	4/163 (2.5%)	1/61 (1.6%)	3/102 (2.9%)	
CB ER status				0.018
ER−	56/163 (34%)	14/61 (23%)	42/102 (41%)	
ER+	107/163 (66%)	47/61 (77%)	60/102 (59%)	
CB PR status				<0.001
PR−	86/163 (53%)	21/61 (34%)	65/102 (64%)	
PR+	77/163 (47%)	40/61 (66%)	37/102 (36%)	
CB HER2 IHC status				<0.001
2+	27/156 (17%)	22/58 (38%)	5/98 (5.1%)	
3+	129/156 (83%)	36/58 (62%)	93/98 (95%)	
Not reported	7	3	4	
CB HER2 FISH ratio	6.8 (3.9) 6.1 [2.0 20.7]	4.9 (2.9) 4.3 [2.0 13.0]	8.1 (4.0) 7.2 [2.5 20.7]	<0.001
Unknown	66	21	45	
CB Ki67	38 (22) 34 [1 98]	36 (21) 34 [1 98]	40 (22) 34 [5 95]	0.3
Unknown	27	6	21	
CB p53	31 (37) 6 [0 99]	41 (40) 21 [0 99]	26 (34) 5 [0 95]	0.092
Unknown	109	44	65	
Neoadjuvant regimen				
ddAC‐THP	6/163 (3.7%)	2/61 (3.3%)	4/102 (3.9%)	
Other	2/163 (1.2%)	1/61 (1.6%)	1/102 (1.0%)	
TCHP	138/163 (85%)	49/61 (80%)	89/102 (87%)	
TDM1 + Pertuzumab	2/163 (1.2%)	1/61 (1.6%)	1/102 (1.0%)	
THP	13/163 (8.0%)	8/61 (13%)	5/102 (4.9%)	
THP followed by ddAC	2/163 (1.2%)	0/61 (0%)	2/102 (2.0%)	
Type of surgery				0.3
Breast conserving surgery	64/163 (39%)	21/61 (34%)	43/102 (42%)	
Total mastectomy	99/163 (61%)	40/61 (66%)	59/102 (58%)	

Abbreviations: CB, core biopsy; ER, estrogen receptor; IHC, immunohistochemistry; pCR, pathologic complete response; PR, progesterone receptor.

^a^ *n*/*N* (%); Mean (SD) Median [Minimum Maximum].

^b^ Mean (SD) Median [Minimum Maximum]; *n*/*N* (%).

^c^ Wilcoxon rank‐sum test; Fisher's exact test; Pearson's Chi‐squared test.

A total of 102 (63%) patients achieved a pCR as defined by ypT0/is and no lymph node metastasis. Among the patients who achieved a pCR, 93 (91%) had IHC 3+ tumors at initial diagnosis. The median HER2 FISH ratio for patients who achieved a pCR was 7.2 (range 2.5–20.7) compared to 4.3 (0.0–13.0) for those with residual disease (*p* < 0.001). Tumor characteristics for those with and without a pCR were statistically significant for nuclear grade (*p* = 0.013), ER status (*p* = 0.018), PR status (*p* < 0.001), and HER2 IHC (*p* < 0.001) (Table 1). Age, clinical T and N classification, ki67, p53, and type of surgery were not statistically different according to pCR status.

Among the 61 patients (37%) who did not achieve a pCR after neoadjuvant therapy, 25 (41%) had HER2‐negative residual disease (Table 2). Age, primary tumor characteristics at diagnosis (clinical T classification, grade, hormone receptor status, ki67, and p53), and pathologic tumor characteristics (pathologic T classification, lymph node status, grade, hormone receptor status, ki67, and p53) did not statistically differ by residual disease HER2 status. HER2 IHC scores before neoadjuvant therapy did not predict for loss or retention of HER2 status (*p* > 0.9). The median baseline FISH ratio value for HER2 concordant disease was 5.15 (2.1–13) and the median baseline FISH ratio value for HER2 discordant disease was 2.46 (2–8.2). Clinical nodal status was statistically different between patients with HER2‐positive tumors after neoadjuvant therapy and HER2‐negative tumors after neoadjuvant therapy (*p* = 0.019). The use of adjuvant chemotherapy and the use of adjuvant anti‐HER2‐targeted therapy were not significantly different according to residual HER2 status (*p* = 0.7 and *p* > 0.9). Almost all patients with residual disease received some form of adjuvant HER2‐targeted therapy, except for one patient with HER2‐positive residual disease and one patient with HER2‐negative residual disease.

**TABLE 2:** Characteristics of patients, tumors, and treatment regimens of patients with residual disease according to residual disease HER2 status after neoadjuvant treatment.

	Whole cohort	Residual disease HER2 status		
	*N* = 61^a^	HER2−, *N* = 25^b^	HER2+, *N* = 36^b^	*p*‐value^c^
Age at diagnosis	49 (13) 48 [26 81]	51 (14) 48 [27 81]	49 (13) 46 [26 77]	0.6
CB histology				
Ductal	57/61 (93%)	22/25 (88%)	35/36 (97%)	
Lobular	2/61 (3.3%)	1/25 (4.0%)	1/36 (2.8%)	
Mixed	1/61 (1.6%)	1/25 (4.0%)	0/36 (0%)	
Unspecified	1/61 (1.6%)	1/25 (4.0%)	0/36 (0%)	
Clinical T classification				0.5
1	7/61 (11%)	2/25 (8.0%)	5/36 (14%)	
2	32/61 (52%)	16/25 (64%)	16/36 (44%)	
3	17/61 (28%)	6/25 (24%)	11/36 (31%)	
4	5/61 (8.2%)	1/25 (4.0%)	4/36 (11%)	
Clinical N classification				0.019
0	23/60 (38%)	13/25 (52%)	10/35 (29%)	
1	30/60 (50%)	10/25 (40%)	20/35 (57%)	
2	2/60 (3.3%)	2/25 (8.0%)	0/35 (0%)	
3	5/60 (8.3%)	0/25 (0%)	5/35 (14%)	
NX	1	0	1	
CB nuclear grade				0.2
2	29/61 (48%)	10/25 (40%)	19/36 (53%)	
2.5	3/61 (4.9%)	0/25 (0%)	3/36 (8.3%)	
3	28/61 (46%)	14/25 (56%)	14/36 (39%)	
Unknown	1/61 (1.6%)	1/25 (4.0%)	0/36 (0%)	
CB ER status				0.4
ER−	14/61 (23%)	7/25 (28%)	7/36 (19%)	
ER+	47/61 (77%)	18/25 (72%)	29/36 (81%)	
CB PR status				0.7
PR−	21/61 (34%)	8/25 (32%)	13/36 (36%)	
PR+	40/61 (66%)	17/25 (68%)	23/36 (64%)	
CB HER2 IHC				>0.9
2+	22/58 (38%)	9/24 (38%)	13/34 (38%)	
3+	36/58 (62%)	15/24 (62%)	21/34 (62%)	
Unknown	3	1	2	
CB HER2 FISH ratio	4.93 (2.91) 4.30 [2.00 13.00]	3.77 (2.18) 2.46 [2.00 8.20]	5.71 (3.12) 5.15 [2.10 13.00]	0.023
Unknown	21	9	12	
CB Ki67	36 (21) 34 [1 98]	37 (25) 34 [1 98]	35 (18) 32 [4 74]	>0.9
Unknown	6	2	4	
CB p53	41 (40) 21 [0 99]	26 (37) 5 [0 99]	59 (39) 70 [0 98]	0.15
Unknown	44	16	28	
Neoadjuvant regimen				
ddAC‐THP	2/61 (3.3%)	0/25 (0%)	2/36 (5.6%)	
Other	1/61 (1.6%)	1/25 (4.0%)	0/36 (0%)	
TCHP	49/61 (80%)	19/25 (76%)	30/36 (83%)	
TDM1 + Pertuzumab	1/61 (1.6%)	1/25 (4.0%)	0/36 (0%)	
THP	8/61 (13%)	4/25 (16%)	4/36 (11%)	
THP followed by ddAC	0/61 (0%)	0/25 (0%)	0/36 (0%)	
Pathologic T classification				0.7
1	50/60 (83%)	20/25 (80%)	30/35 (86%)	
2	9/60 (15%)	4/25 (16%)	5/35 (14%)	
3	1/60 (1.7%)	1/25 (4.0%)	0/35 (0%)	
Unknown	1	0	1	
0	0/61 (0%)	0/25 (0%)	0/36 (0%)	
Positive lymph node at surgery				0.4
No	38/61 (62%)	17/25 (68%)	21/36 (58%)	
Yes	23/61 (38%)	8/25 (32%)	15/36 (42%)	
ER status after NAT				0.2
ER−	10/61 (16%)	2/25 (8.0%)	8/36 (22%)	
ER+	51/61 (84%)	23/25 (92%)	28/36 (78%)	
PR status after NAT				0.13
PR−	29/61 (48%)	9/25 (36%)	20/36 (56%)	
PR+	32/61 (52%)	16/25 (64%)	16/36 (44%)	
HER2 IHC after NAT				<0.001
0	3/61 (4.9%)	3/25 (12%)	0/36 (0%)	
1+	10/61 (16%)	8/25 (32%)	2/36 (5.6%)	
2+	30/61 (49%)	14/25 (56%)	16/36 (44%)	
3+	18/61 (30%)	0/25 (0%)	18/36 (50%)	
HER2 FISH ratio after NAT	3.75 (2.91) 2.80 [1.00 13.20]	1.28 (0.31) 1.20 [1.00 1.90]	5.28 (2.74) 4.56 [1.58 13.20]	<0.001
Unknown	14	7	7	
Nuclear grade after NAT				>0.9
1	7/47 (15%)	3/19 (16%)	4/28 (14%)	
2	31/47 (66%)	13/19 (68%)	18/28 (64%)	
2.5	1/47 (2.1%)	0/19 (0%)	1/28 (3.6%)	
3	8/47 (17%)	3/19 (16%)	5/28 (18%)	
Unknown	14	6	8	
Ki67 after NAT	14 (19) 6 [0 73]	11 (16) 5 [1 73]	15 (20) 8 [0 73]	0.8
Unknown	7	4	3	
p53 after NAT	25 (37) 8 [0 98]	17 (31) 5 [0 97]	34 (42) 10 [0 98]	0.4
Unknown	43	16	27	
Adjuvant chemotherapy				0.7
No	40/61 (66%)	17/25 (68%)	23/36 (64%)	
Yes	21/61 (34%)	8/25 (32%)	13/36 (36%)	
Adjuvant HER2‐targeted treatment				>0.9
No	2/61 (3.3%)	1/25 (4.0%)	1/36 (2.8%)	
Yes	59/61 (97%)	24/25 (96%)	35/36 (97%)	
Adjuvant trastuzumab				0.7
No	9/61 (15%)	3/25 (12%)	6/36 (17%)	
Yes	52/61 (85%)	22/25 (88%)	30/36 (83%)	
Adjuvant pertuzumab				0.8
No	38/61 (62%)	16/25 (64%)	22/36 (61%)	
Yes	23/61 (38%)	9/25 (36%)	14/36 (39%)	
Adjuvant trastuzumab emtansine				0.5
No	46/61 (75%)	20/25 (80%)	26/36 (72%)	
Yes	15/61 (25%)	5/25 (20%)	10/36 (28%)	
Adjuvant neratinib				0.6
No	56/61 (92%)	24/25 (96%)	32/36 (89%)	
Yes	5/61 (8.2%)	1/25 (4.0%)	4/36 (11%)	
Adjuvant tucatinib				>0.9
No	59/61 (97%)	24/25 (96%)	35/36 (97%)	
Yes	2/61 (3.3%)	1/25 (4.0%)	1/36 (2.8%)	
Adjuvant lapatinib				>0.9
No	60/61 (98%)	25/25 (100%)	35/36 (97%)	
Yes	1/61 (1.6%)	0/25 (0%)	1/36 (2.8%)	
Adjuvant trastuzumab deruxtecan				0.5
No	59/61 (97%)	25/25 (100%)	34/36 (94%)	
Yes	2/61 (3.3%)	0/25 (0%)	2/36 (5.6%)	
Adjuvant trastuzumab‐anns				>0.9
No	60/61 (98%)	25/25 (100%)	35/36 (97%)	
Yes	1/61 (1.6%)	0/25 (0%)	1/36 (2.8%)	

Abbreviations: CB, core biopsy; ER, estrogen receptor; IHC, immunohistochemistry; NAT, neoadjuvant therapy; PR, progesterone receptor.

^a^ *n*/*N* (%); Mean (SD) Median [Minimum Maximum].

^b^ Mean (SD) Median [Minimum Maximum]; *n*/*N* (%).

^c^ Wilcoxon rank‐sum test; Fisher's exact test; Pearson's Chi‐squared test.

**FIGURE 2:**
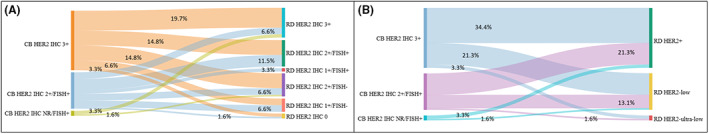
Change in HER2 expression following neoadjuvant treatment. The Sankey diagrams show the change in HER2 expression according to IHC and/or FISH results (panel A) and classification by HER2‐low and HER2‐ultralow expression (panel B). CB, core biopsy; NR, not recorded; RD, residual disease.

The change of HER2 expression from the initial core biopsy to residual disease after neoadjuvant dual HER2‐targeted therapy plus chemotherapy using the new categories of HER2‐low and HER2‐ultralow is shown in Figure 2. Of the 36 patients with HER2 IHC 3+ on the initial core biopsy, 12 remained HER2 IHC 3+, nine evolved to HER2 IHC 2+ with HER2 gene amplification by FISH, nine evolved to HER2 IHC 2+ without HER2 gene amplification by FISH, four evolved to HER2 IHC 1+, and two evolved to HER2 IHC 0. Of the 22 patients with HER2 IHC 2+ with HER2 gene amplification by FISH on the initial core biopsy, seven remained HER2 IHC 2+ with HER2 gene amplification by FISH, four evolved to HER2 IHC 3+, four evolved to HER2 IHC 2+ without HER2 gene amplification by FISH, four evolved to HER2 IHC 1+ without HER2 gene amplification by FISH, two evolved to HER2 IHC 1+ with HER2 gene amplification by FISH, and one evolved to HER2 IHC 0. Lastly, of the three patients with HER2 IHC not reported (NR) with HER2 gene amplification by FISH on the initial core biopsy, two evolved to HER2 IHC 3+ and one evolved to HER2 IHC 2+ without HER2 gene amplification by FISH. Overall, of the 61 HER2‐positive patients with residual disease after neoadjuvant therapy, 36 (59%) patients had HER2‐positive residual disease, 24 (36%) had HER2‐low residual disease, and three (4.9%) had HER2‐ultralow residual disease. Of the 25 patients with HER2‐negative residual disease using the traditional definition, 88% were classified as HER2‐low, while 12% were classified as HER2‐ultralow.

With respect to ER status discordance, there were a total of six ER discordant patients. Of the 47 patients with residual disease whose tumors were ER positive on the core biopsy, one became negative after neoadjuvant treatment. Of the 14 patients with residual disease whose tumors were ER negative on the core biopsy, five became positive after neoadjuvant treatment. In these six patients with ER discordance, five lost their HER2‐positivity status. In the 55 who were ER concordant, 20 lost their HER2‐positivity status.

**FIGURE 3:**
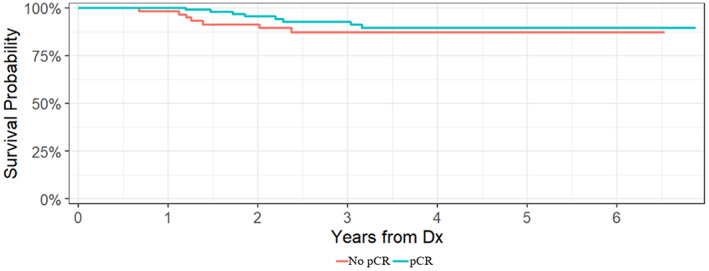
Invasive disease‐free survival by pCR status.

**FIGURE 4:**
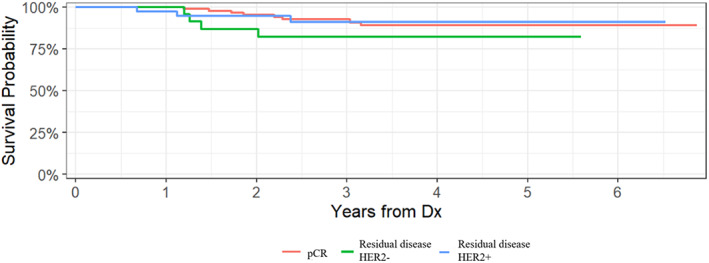
Invasive disease‐free survival by residual disease HER2 status after neoadjuvant treatment.

With a median follow‐up of 3.3 years (min 0.42 to max 6.87 years), there were 15 IDFS events and three deaths in the entire cohort. Each of the patients who died had a previous recurrence. In the 61 patients with residual disease at the time of surgery, there were seven IDFS events and two deaths. Specifically, there were three recurrences among the patients with HER2 concordance, four recurrences among the patients with HER2 discordance, and one death in each group. Overall, the 3‐year IDFS rate for the entire cohort was 91% (95% confidence interval [CI], 86–96%). Patients with a pCR had a 3‐year IDFS rate of 93% (95% CI, 87–99%) compared to a 3‐year IDFS rate of 87% (95% CI, 79–97%) for patients with residual disease. Patients who retained HER2‐positivity after neoadjuvant treatment had a 3‐year IDFS rate of 91% (95% CI, 91%–100%), while patients who lost HER2‐positivity had a 3‐year IDFS rate of 82% (95% CI, 67%–100%). Kaplan–Meier curves for IDFS according to pCR status and residual disease HER2 status after neoadjuvant treatment are demonstrated in Figures 3 and 4.

## 4 DISCUSSION

In this retrospective study of 163 patients with HER2‐positive early stage breast cancer, the majority (63%) experienced a pCR with neoadjuvant chemotherapy plus dual HER‐directed therapy. A total of 91% of patients with pCR had HER2 3+ by IHC which emphasizes the strong association of IHC 3+ in predicting pCR. Among the 37% of patients who did not achieve a pCR after neoadjuvant therapy, 41% had HER2‐negative residual disease. Although the HER2 IHC staining pattern did not predict for loss of HER2, the median baseline FISH ratio values for HER2‐concordant disease were higher than that for HER2‐discordant disease. This suggests that increased HER2 FISH ratios may predict for retention of HER2‐positivity following neoadjuvant treatment.

Several studies that have investigated the impact of neoadjuvant HER2‐targeted treatment with trastuzumab alone plus chemotherapy report a HER2 discordance rate ranging from 7% to 43% from the time of core biopsy to surgery.^7^, ^8^, ^9^, ^10^, ^11^, ^12^, ^13^, ^14^, ^15^, ^16^, ^17^, ^18^, ^19^ However, there are few studies that report the discordance rate after neoadjuvant dual HER2‐targeted treatment plus chemotherapy, the current standard‐of‐care. In one retrospective analysis of 130 patients treated with neoadjuvant dual HER2‐targeted therapy plus chemotherapy, Ferraro et al. reported a HER2 discordance rate of 52%, although only 25 patients were eligible for analysis.^25^ In another retrospective study of 500 HER2‐positive breast cancer patients, Katayama et al. reported an overall discordance rate of 22.3% after neoadjuvant treatment.^26^ However, only 8.8% of patients received dual HER2‐targeted treatment, and a percentage of patients did not receive any HER2‐targeted therapy or chemotherapy. In a retrospective cohort study of 348 HER2‐positive breast cancer patients, of which 58.9% received neoadjuvant dual HER2‐targeted treatment, Wetzel et al. reported an overall discordance rate of 28%. In patients who received neoadjuvant TCHP, the discordance rate was 34.4%, whereas the discordance rate was 28.1% for patients who received TCH.^20^ In addition, Ignatov and colleagues performed a retrospective study of 205 patients with HER2‐positive breast cancer with residual disease—of which 167 received neoadjuvant single‐agent HER2‐targeted therapy and 19 received dual anti‐HER2 treatment—and reported an overall discordance rate of 42%.^13^ On subgroup analyses by specific HER2‐targeted treatment, the discordance rate with trastuzumab alone was 47.3%, whereas the discordance rate with dual‐agent trastuzumab and pertuzumab was 63.2%. However, this subgroup analysis was limited by size given that only 19 patients received dual anti‐HER2 treatment. Notably, 21 (10.7%) patients in this study did not receive chemotherapy with HER2‐directed therapy in the neoadjuvant regimen. Lastly, Branco and colleagues performed a retrospective study of 108 HER2‐positive breast cancer patients and reported a HER2 discordance rate of 13.3% in patients treated with neoadjuvant therapy with residual disease; however, only three patients (including those with a pCR) received neoadjuvant dual HER2‐targeted treatment.^8^

A subgroup analysis of the phase 3, randomized, multicenter KATHERINE study, which investigated adjuvant T‐DM1 versus trastuzumab in patients with residual disease after neoadjuvant taxane and trastuzumab therapy, was performed to investigate outcomes of T‐DM1 and trastuzumab based on loss of HER2‐positivity at the time of surgery.^27^ In the KATHERINE trial, patients were allowed to complete any neoadjuvant chemotherapy regimen with at least 9 weeks of taxane‐based therapy and 9 weeks of trastuzumab therapy. In the subgroup analysis which included 845 patients with paired tumor samples available from the pre‐neoadjuvant therapy core biopsy and surgical tumor specimen with HER2 status of the residual disease known, 8.3% of tumors were HER2‐negative after neoadjuvant treatment. In the KATHERINE trial, 290 (19.5%) of the 1486 participants received dual HER2‐directed neoadjuvant therapy with trastuzumab plus any additional HER2‐targeted agent, and in the subgroup analysis, 160 (18.9%) of the 845 patients received dual HER2‐targeted treatment with trastuzumab plus any additional HER2‐targeted agent. Of these 160 patients who received dual HER2‐targeted treatment, 21 (13.1%) were HER2‐negative at the time of surgery.^27^

The HER2 discordance rate in our study may differ from the previously mentioned studies due to different neoadjuvant regimens used and various definitions of HER2‐positivity. Specifically, each of the studies included different percentages of patients who received dual HER2‐targeted therapy and different percentages of patients who received chemotherapy in the neoadjuvant regimen. In addition, different chemotherapy regimens were used. Moreover, various definitions of HER2‐positivity were used which may lead to an over‐ or underestimate of HER2 discordance. Our study used the definition of HER2‐positivity most similar to that used in the KATHERINE trial. Lastly, IHC can be subjective depending on the pathologist reading the biopsy, which can also cause variability in HER2‐positivity rates.

The mechanism of HER2 discordance with dual‐agent HER2‐targeted treatment compared to trastuzumab alone is not well‐understood. It has been shown that combination therapy with trastuzumab and pertuzumab results in increased disruption of HER2 receptor dimers and leads to a dose‐dependent downregulation of HER2 receptor levels, which may result in reduced HER2 expression.^8^ In addition, given that trastuzumab and pertuzumab results in a synergistic effect on increased breast cancer cell death^8^ and that intratumoral heterogeneity of HER2 gene expression exists,^28^ treatment with dual‐agent HER2‐targeted therapy may result in increased clonal selection for HER‐negative tumor cells.

Given the small number of IDFS events and deaths in our study and short follow‐up time, survival analyses were limited. Nevertheless, it appears that clinically meaningful differences in IDFS were not observed for patients with HER2 concordant disease and HER2 discordant disease after neoadjuvant treatment. Previous studies are inconsistent regarding whether the loss of HER2 amplification after neoadjuvant treatment is associated with worse outcomes.^7^, ^8^, ^9^, ^11^, ^12^, ^13^, ^14^, ^15^, ^17^, ^18^, ^19^, ^20^ For example, Branco et al. report the 5‐year IDFS and 5‐year OS of patients who retained HER2 amplification after neoadjuvant treatment is 70% and 84%, respectively, and 21% and 50% for patients whose residual tumors lost HER2 amplification (*p* = 0.02 and *p* < 0.001).^8^ In contrast, Wetzel et al. found no difference in 5‐year recurrence‐free survival and OS for patients with HER2‐positive residual disease compared to patients with HER2‐negative residual disease.^20^ A possible reason for the discrepancy of studies with respect to whether loss of HER2 status predicts for worse survival may be due to studies being underpowered to detect a significance. Studies also have different follow‐up times and different proportion of patients who received adjuvant HER2‐targeted treatment and adjuvant chemotherapy, which can each impact long‐term survival outcomes.

Given that the adjuvant treatment regimen is currently determined by the HER2 status prior to neoadjuvant treatment, this study raises the question of whether repeat HER2 testing on residual tumor may change treatment. In our study, there was no difference in the use of chemotherapy or adjuvant HER2‐targeted treatment in patients whose residual tumors retained HER2 amplification compared to those who lost HER2 amplification, which suggests that the loss of HER2 status did not impact the decision to administer adjuvant HER2‐targeted therapy. In the subgroup analysis of the KATHERINE trial which investigated T‐DM1 versus trastuzumab in patients with HER2‐negative disease at surgery, there were no IDFS events among patients randomized to T‐DM1 (*n* = 28) compared to 11 events in patients randomized to trastuzumab (*n* = 42), which suggests that T‐DM1 is effective for patients with HER2‐negative residual disease.^27^ However, this analysis was limited by small sample size and the fact that only 18.9% of patients in this subgroup received dual HER2‐targeted treatment.

In addition, our study showed that almost all patients (except for three) with HER2‐negative residual disease have tumors that can be classified as HER2‐low. Miglietta et al. reported similar results in a study of 446 HER2‐positive and HER2‐negative breast cancer patients evaluating HER2‐low expression after neoadjuvant treatment.^14^ In this study, 7% of patients with HER2‐positive breast cancer lost HER2‐positivity, with all patients converting to HER2‐low after neoadjuvant treatment and none converting to HER2 IHC 0. However, the percentage of patients who lost HER2‐positivity may differ from that described in our study, given that not all patients received neoadjuvant HER2‐targeted therapy. Nevertheless, both studies demonstrate that nearly all patients who lose HER2‐positivity after neoadjuvant treatment evolve to HER2‐low, which indicates that most tumors retain some low level of HER2 expression following neoadjuvant treatment. Given that T‐DXd has shown effectiveness in HER2‐low metastatic breast cancer, the results of our study raise the question of whether this subset of patients whose tumors evolve to HER2‐low may derive a greater benefit from T‐DXd than T‐DM1.^22^ The results of the phase 3, multicenter, randomized DESTINY‐Breast05 study (ClinicalTrials.gov identifier: NCT04622319) investigating trastuzumab deruxtecan versus T‐DM1 as adjuvant therapy in patients with HER2‐positive early breast cancer will hopefully shed some light on this issue.

Our study has several limitations. First, it is a modestly powered, retrospective, single institution study with a relatively small sample size (*n* = 163). Second, patients were excluded if hormone receptor profiles were not performed on residual tumors. This most often occurred in patients with microscopic tumors at surgery, which may have been tumors that retained HER2 status given their response to treatment, causing the HER2 discordance rate to be an overestimate. In addition, because patients were included in this study based on available pathology reports, this allowed patients to be included in this study who had surgeries and pathology review performed by an outside institution. Therefore, central repeat testing was not consistently performed for HER2 in the residual tumor and HER2 was not reread to confirm agreement with HER2 loss. Finally, there were a limited number of recurrence events and deaths which limited the survival analysis.

In conclusion, our results show that 41% of HER2‐positive breast cancers become HER2‐negative after neoadjuvant dual‐agent HER2‐targeted therapy with chemotherapy. Of these patients, 88% were classified as HER‐low. Although our study was limited by a small number of recurrences and deaths and a short follow‐up time, there appears to be no clinically meaningful differences in IDFS between patients who lost and retained HER2‐positivity. Further research is needed to determine the most appropriate adjuvant treatment regimen for these high‐risk patients with residual disease who lose HER2‐positivity after neoadjuvant treatment.

## AUTHOR CONTRIBUTIONS

**Alexis LeVee:** Conceptualization (equal); data curation (equal); formal analysis (equal); investigation (lead); methodology (equal); project administration (lead); resources (equal); visualization (equal); writing – original draft (lead); writing – review and editing (lead). **Kellie Spector:** Conceptualization (equal); data curation (equal); investigation (supporting); methodology (supporting); project administration (supporting); resources (equal); writing – review and editing (equal). **Brigid Larkin:** Data curation (supporting); investigation (supporting); writing – review and editing (equal). **Felipe Dezem:** Formal analysis (equal); methodology (equal); resources (equal); software (equal); writing – review and editing (equal). **Jasmine Plummer:** Formal analysis (equal); methodology (equal); resources (equal); software (equal); writing – review and editing (equal). **Farnaz Dadmanesh:** Validation (equal); writing – review and editing (equal). **Sujata Patil:** Formal analysis (equal); methodology (equal); resources (equal); software (equal); writing – review and editing (equal). **Heather L. McArthur:** Conceptualization (equal); data curation (equal); methodology (equal); project administration (equal); resources (equal); supervision (lead); writing – review and editing (equal).

## FUNDING INFORMATION

None.

## CONFLICT OF INTEREST STATEMENT

Heather L. McArthur has consulted for Amgen, Bristol‐Myers Squibb, Celgene, Eli Lilly, Genentech/Roche, Immunomedics/Gilead, Merck, OBI Pharma, Pfizer, Puma, Spectrum Pharmaceuticals, Syndax Pharmaceuticals, Peregrine, Calithera, Daiichi‐Sankyo, Seattle Genetics, AstraZeneca, and TapImmune; and has received research support from Bristol‐Myers Squibb; MedImmune, LLC/AstraZeneca; BTG; and Merck. The other authors have no conflicts to disclose.

## Data Availability

The data that support the findings of this study are available from the corresponding author upon reasonable request.
